# Prevalence and genotype distribution of hepatitis C virus within hemodialysis units in Thailand: role of HCV core antigen in the assessment of viremia

**DOI:** 10.1186/s12879-022-07074-2

**Published:** 2022-01-22

**Authors:** Natthaya Chuaypen, Apichaya Khlaiphuengsin, Thaninee Prasoppokakorn, Paweena Susantitaphong, Wisit Prasithsirikul, Anchalee Avihingsanon, Pisit Tangkijvanich, Kearkiat Praditpornsilpa

**Affiliations:** 1 Center of Excellence in Hepatitis and Liver Cancer, Department of Biochemistry, Faculty of Medicine, Chulalongkorn University, Bangkok, 10330 Thailand; 2 Division of Gastroenterology, Department of Medicine, Faculty of Medicine, Chulalongkorn University, Bangkok, Thailand; 3 Division of Nephrology, Department of Medicine, Faculty of Medicine, Chulalongkorn University and King Chulalongkorn Memorial Hospital, Bangkok, Thailand; 4 Department of Disease Control, Bamrasnaradura Infectious Diseases Institute, Nonthaburi, Thailand; 5 HIV-NAT, Thai Red Cross AIDS Research Centre, Bangkok, Thailand

**Keywords:** HCV, Genotype, Core antigen, Transmission, Hemodialysis, Dialyzer reuse

## Abstract

**Background:**

Individuals with end-stage renal disease have a higher risk of hepatitis C virus (HCV) acquisition during long-term hemodialysis (HD). Our report was designed to investigate HCV prevalence and genotype, in addition to the clinical use of HCV core antigen (HCVcAg), within multiple HD facilities in Thailand.

**Methods:**

This cross‐sectional report was investigated between January and June 2019. HCV infection was assessed by anti-HCV and confirmed active infection by measuring HCV RNA and HCVcAg. HCV genotype was determined by phylogenetic analysis using nucleotide sequences of NS5B region.

**Results:**

Overall, 140 of 3,305 (4.2%) patients in 15 dialysis centers had anti-HCV positive. Among them, HCV RNA was further assessed in 93 patients and was detectable in 59 (63.4%) persons. Considering HCV viremia, HCVcAg measurement exhibited high accuracy (96.8%), sensitivity (94.9%) and specificity (100%) in comparison with HCV RNA testing. Moreover, individuals infected with HCV received a longer duration of dialysis vintage when compared to anti-HCV negative controls. The major sub-genotypes were 1a, 1b, 3a, 3b, 6f and 6n. Regarding phylogenetic analysis, there were 7 clusters of isolates with high sequence homology affecting 17 individuals, indicating possible HCV transmission within the same HD centers.

**Conclusions:**

HCV frequency and common sub-genotypes in HD centers were different from those found in the Thai general population. HCVcAg might be an alternate testing for viremia within resource-limited countries. Enhanced preventive practices, dialyzer reuse policy and better access to antiviral therapy are crucial for HCV micro-elimination within HD facilities.

## Background

Hepatitis C virus (HCV) infection is an important global health problem, with approximately 71 million people are chronically infected with the virus [1]. HCV has been classified into seven major genotypes, each containing a variable number of closely related subtypes [2]. In Thailand, the prevalence of HCV in the general population is approximately 1%, with the most common genotypes are genotype 3 (HCV-3), followed by genotype 1 (HCV-1) and genotype 6 (HCV-6) [3]. Chronic HCV infection is a major etiological cause of chronic hepatitis, cirrhosis, hepatic decompensation and hepatocellular carcinoma (HCC). Moreover, several extrahepatic manifestations have been shown to be associated to chronic HCV infection including insulin resistance, type 2 diabetes, cardiovascular diseases and chronic kidney disease (CKD) [4]. A meta-analysis has indicated that chronic HCV infection is linked to 50% increased risk of proteinuria and 40% increased incidence of CKD [5]. Moreover, HCV-infected patients with CKD tend to have an accelerated rate of renal impairment leading to progressive end-stage renal disease (ESRD) and increased related-complications [6]. With the advance of interferon-free direct-acting antivirals (DAAs), HCV therapy has been substantially changed as difficult-to-treat populations including patients with late-stage CKD could achieve sustained virological response (SVR) rates over 90% [7]. Current data have demonstrated that successful HCV antiviral therapy not only reduces the risk of cirrhosis and HCC but also improves extrahepatic manifestations of HCV infection [7].

As HCV transmission occurs primarily through blood-to-blood contact, patients with CKD stages 5 undergoing maintenance hemodialysis (HD) have an increased risk of HCV infection. Despite a trend toward lower HCV prevalence among general populations, the infected rate in patients with late-stage CKD continues to be a major concern [6]. Such higher prevalence has been linked to an increased exposure for HCV acquisition including frequent invasive medical procedures and nosocomial transmission within HD units [8]. Moreover, the prevalence of HCV infection in patients with CKD undergoing HD differ significantly across global regions, ranging from approximately 1.5–30%, and 5–40%, in developed and developing countries, respectively [9]. Additionally, isolated small-scale HCV outbreaks (≥ 2 patients) in HD units still occur sporadically worldwide even with available specific infection control guidelines [8]. These data highlight the need for better assessment of the infection, improved infection control measures and increased access to antiviral treatments to prevent potential spreading of HCV in HD settings.

According to the Kidney Disease: Improving Global Outcome (KDIGO) guideline [10], it is recommended that all patients with CKD should undergo testing for HCV infection before initiating dialysis. In this context, screening of anti-HCV antibodies by immunoassays and confirmation of active HCV infection by nucleic acid testing (NAT) are necessary. Moreover, patients in dialysis units should be regularly tested with serum alanine aminotransferase (ALT) monthly and anti-HCV antibodies or NAT for HCV RNA semi-annually. Recently, serum HCV core antigen (HCVcAg) quantification has emerged as an alternative assay to HCV RNA quantification because of its rapidity, being less expensive and more feasibility in resource-limited settings [11]. In addition, a recent report has shown that HCVcAg measurement is cost-saving and could potentially replace HCV RNA for detecting HCV viremia [12]. To date, data regarding the utility of serum HCVcAg in monitoring active HCV infection in patients with CKD are inadequate.

The aims of this study were to investigate the prevalence and genotype distribution of HCV in patients undergoing long-term HD across several centers in Thailand. The possibility of patient-to-patient transmission of HCV within the same HD facilities was also determined using phylogenetic analysis. In addition, this study evaluated the usefulness of serum HCVcAg as a potential replacement of HCV RNA in assessing HCV viremia. Finally, we proposed a simple algorithm for the detection and monitoring of HCV infection among patients undergoing HD.

## Methods

### Participants

A cross‐sectional study was performed among patients undergoing chronic HD at 15 centers in Bangkok and other provinces from January to June 2019. Screening for HCV infection in these centers was carried out using anti-HCV antibody. Patients with positive results in the screening assays were invited to participate in the study and further assessed for HCV viremia and molecular analysis. Patients co-infected with hepatitis B virus (HBV) or human immunodeficiency virus (HIV) were excluded.

### Laboratory assays

Serum aspartate aminotransferase (AST), alanine aminotransferase (ALT) and other biochemical tests were evaluated by standard methods. AST/platelet ratio index (APRI) as a non-invasive serum marker for liver fibrosis was calculated by the following formula: (AST/upper limit of normal considered as 40 IU/L)/platelet count (10^9^/L) × 100. In a meta-analysis, an APRI score greater than 1.0 had a sensitivity and specificity of 76% and 72%, respectively for predicting cirrhosis. In addition, an APRI score greater than 0.7 had a sensitivity of 77% and specificity of 72% for predicting significant fibrosis [13].

Anti-HCV antibody was tested with a third-generation chemiluminescent microparticle immunoassay (CMIA, ARCHITECT system, Abbott Diagnostics, Wiesbaden, Germany). HCV RNA quantification was performed using real-time quantitative reverse-transcription polymerase chain reaction (RT-PCR) (Abbott Molecular Inc. Des Plaines, IL, USA) in accordance with the manufacturer’s instructions. The lower and upper detection limits of the assay were < 12 IU/mL and 100,000,000 IU/mL, respectively. Serum HCVcAg quantification was analyzed by a two-step CMIA (Abbott Diagnostics, Tokyo, Japan). The test allows the determination of HCVcAg in a linear range 3–20,000 femtomoles/liter (fmol/L). Samples with concentrations between 3 and 10 fmol/L were defined as ‘grey zone’ and were re-examined in duplicate as manufacturer recommendations [14]. If at least one duplicate test was positive or in grey-zone, the sample was considered as positive test.

### HCV genotyping

HCV genotyping was performed as previously described [3]. Briefly, HCV RNA was extracted from anti-HCV positive serum samples by guanidine thiocyanate method. cDNAs were generated from viral RNA by using RevertAid first strand cDNA synthesis kit (Thermo Scientific) according to the manufacturer’s instructions. Genotype was determined based on the nucleotide sequence of the NS5B region [15, 16]. Nested PCR of the NS5B region was performed by using primer pairs of NS5B_F1 and NS5B_R1 in the first round and NS5B_F2 and NS5BR2 were used in the second round. First and second amplification reactions were as followed: pre-incubation at 94 °C for 3 min., 40 cycles of denaturation at 94 °C for 1 min., annealing at 52 °C for 1 min., extension at 72 °C for 1.30 min. and a final extension step at 72 °C for 7 min. The sequences were edited using Bio-Edit (v.7.2.5) (Ibis Therapeutics, Carlsbad, CA) and similarity between sequences was examined by the BLASTN program (http://www.ncbi.nlm.nih.gov).

### Phylogenetic analysis

Multiple alignments were performed using CLUSTALW (Bio-Edit version 7.2.5 software) and the phylogenic trees of NS5B gene was constructed with matrix of pairwise distances estimated under the maximum-likelihood method based on the general time-reversible (GTR) substitution, gamma-distribution (G) and invariant sites(I); GTR + G + I model [17] by using MEGA X 10.2.1 software. The statistical analysis of constructed trees was performed by 1,000 replicates bootstrap test to confirm the reliability of the phylogenetic tree. The transmission clusters were identified at a branch support threshold of 0.7 and a genetic distance threshold of 0.045 using Cluster Picker software as described previously [18].

The reference sequences of HCV were retrieved from GenBank as follows: genotype 1a (D10749), genotype 1b (D90208, M58335), genotype 1c (D14853), genotype 2a (AB047639, D00944), genotype 2b (AB030907, D10988), genotype 2c (D50409), genotype 3a (D17763, D28917), genotype 3b (D49374), genotype 5a (AF064490), genotype 6a (AY859526), genotype 6b (D84262), genotype 6c (EF424629), genotype 6d (D84263), genotype 6e (DQ314805), genotype 6f (DQ835760), genotype 6 g (D63822), genotype 6 h (D84265), genotype 6i (DQ835770), genotype 6j (DQ835769), genotype 6 k (D84264), genotype 6 m (DQ835767), genotype 6n (DQ278894, DQ835768), genotype 6r (EU408328), genotype 6t (EF632071, EU246939), genotype 6u (EU246940, EU408330, EU408332), genotype 6v (EU158186, EU798760), genotype 6w (DQ278892, EU643834), genotype 6 s (EU408329).

The nucleotide sequences of NS5B gene have been submitted to the GenBank database under accession numbers MT428260-MT428322.

### Ethics statement

All patients gave their informed written consent. The study was reviewed and approved by the Institutional Review Board, Faculty of Medicine, Chulalongkorn University. The study was performed in accordance with Declaration of Helsinki for the participation of human individuals. Information regarding demographic data were collected using a standardized questionnaire.

### Statistical analyses

Data were expressed as percentages or median with interquartile range (IQR). Comparisons between groups were analyzed by the χ^2^ or Fisher’s exact test for categorical variables and by two-sample *t* tests for continuous variables as appropriate. Correlations between parameters were assessed by the Spearman's rank test. Sensitivity, specificity, positive predictive value (PPV), negative predictive value (NPV) and diagnostic accuracy were calculated in accordance with standard methods. *P* value < 0.05 was considered as a statistical significance. Statistical analyses were performed by the IBM SPSS software version 23.0 (IBM, Chicago, IL, USA).

## Results

### Characteristics of the Participants

Among 3,305 patients who were on chronic HD in the participating centers during the time of the study, 140 (4.2%) individuals were tested positive for anti-HCV antibodies. Among these, 93 (66.4%) patients accepted to participate in the study. The median age of these 93 patients was 54.0 years with the majority were male (67.7%). To compare clinical characteristics and possible risk factors of HCV infection, additional 93 subjects with matched age and gender, randomly selected from the pool of ESRD patients who had anti-HCV negative, were used as the control group (Table 1). Compared to the control group, patients with anti-HCV positive had had a significantly longer dialysis vintage (median of 7.1 years vs. 5.1 years for the anti-HCV negative group, *P* = 0.013). In addition, patients with anti-HCV positive had increased AST and ALT levels, as well as significantly higher APRI scores, representing higher degree of liver fibrosis compared with the control group. Of note, there were 9 (9.7%) and 7 (7.5%) patients with anti-HCV positive, who had an APRI score greater than 0.7 and 1.0, respectively. In contrast, no patients in the anti-HCV negative group had an APRI score greater than 0.7.

**Table 1:** The characteristics of patient in this study

	Total (n = 186)	Patients with anti-HCV positive (n = 93)	Patients with anti-HCV negative (n = 93)	*P*
Age (year)	53.3 (44.8–62.3)	54.0 (44.8–62.6)	52.7 (45.0–62.0)	0.415
Sex				0.358
Male	120 (64.5)	63 (67.7)	57 (61.3)	
Female	66 (35.5)	30 (32.3)	36 (38.7)	
BMI (kg/m^2^)	22.0 (20.1–24.6)	22.1 (19.3–24.5)	21.9 (20.2–24.8)	0.206
IDU	20 (10.8)	15 (16.1)	5 (5.4)	0.031*
MSM	6(3.2)	2(2.2)	4 (4.3)	0.682
Dialysis vintage (years)	7.0 (4.0–10.3)	7.1 (4.0–13.0)	5.1 (4.0–9.0)	0.013*
ALT (U/L)	16.0 (11.3–23.0)	19.5 (14.0–27.5)	14.0 (11.0–21.0)	0.001*
AST (U/L)	18.5 (13.0–26.3)	24 (17.0–32.5)	16 (12.0–21.0)	< 0.001*
Cr (mg/dL)	9.2 (7.2–11.2)	9.1 (6.9–11.1)	9.3 (7.2–11.4)	0.655
APRI	0.23 (0.16–0.32)	0.29 (0.20–0.42)	0.20 (0.14–0.29)	< 0.001*

Data are expressed as median (IQR) or n (%), as appropriate; *P < 0.05

### HCV RNA and HCVcAg testing

Among patients with anti-HCV positivity, HCV RNA quantification was further performed. Fifty-nine of 93 (63.4%) patients displayed active HCV infection with the median HCV RNA level of 5.7 log_10_ IU/mL (range, 2.3–7.5 log_10_ IU/mL), while 34 (36.6%) individuals had undetectable viral load (HCV RNA < 12 IU/mL). Clinical characteristics and risk factors between these groups were compared as shown in Table 2. There was no significant difference between groups in terms of age, gender distribution, GFR and APRI score. However, patients with viremia exhibited significantly higher AST level compared with those with undetectable HCV RNA.

**Table 2:** The characteristics of patients with anti-HCV positive

	Total (n = 93)	Patients with HCV RNA positive (n = 59)	Patients with HCV RNA negative (n = 34)	*P*
Age (year)	54.0 (44.8–62.6)	56.3 (47.5–63.6)	53.2 (43.0–59.9)	0.153
Sex				0.634
Male	63 (67.7)	41 (69.5)	22 (64.7)	
Female	30 (32.3)	18 (30.5)	12 (35.5)	
BMI (kg/m^2^)	22.1 (19.3–24.5)	21.8 (18.5–23.8)	22.5 (19.8–25.8)	0.307
IDU	15 (16.1)	11(18.6)	4 (11.8)	0.560
MSM	2(2.2)	1(1.7)	1 (2.9)	1.000
Dialysis vintage	7.0 (4.0–13.0)	7.0 (5.0–13.0)	7.5 (3.8–13.0)	0.575
ALT (U/L)	19.5 (14.0–27.5)	22 (16–38)	17 (13–23.5)	0.072
AST (U/L)	24 (17–32.5)	26.5 (17.3–36.8)	20 (14–26.5)	0.039*
Cr (mg/dL)	9.2 (6.9–11.1)	9.4 (6.7–11.1)	8.7 (7.3–11.3)	0.597
APRI	0.29 (0.20–0.42)	0.31 (0.22–0.61)	0.26 (0.18–0.33)	0.122

Data are expressed as median (IQR) or n (%), as appropriate; *P < 0.05

To evaluate the usefulness of serum HCVcAg, serum samples of all 93 patients with anti-HCV positive were tested for serum HCV RNA and HCVcAg in parallel. Overall, log_10_HCVcAg levels were strongly correlated with corresponding log_10_HCV RNA levels (r = 0.956, *P* < 0.001). Moreover, there was a high concordance of detectable or undetectable HCVcAg and HCV RNA (Table 3). In our cohort, 3 patients with detectable HCV RNA were negative for HCVcAg, all of whom had low viral load (HCV RNA < 3,000 IU/ml). Using HCV RNA as the gold standard method, HCVcAg demonstrated a high sensitivity (94.9%) specificity (100%), PPV (100%), NPV (91.9%) and diagnostic accuracy (96.8%).

**Table 3:** Comparison between HCVcAg and HCV RNA assays

HCVcAg	HCV RNA		Total
	Positive	Negative	
Positive	56	0	56
Negative	3	34	37
Total	59	34	93

### HCV genotypes and phylogenetic analysis

A total of 56 out of 59 (94.9%) sera from patients with HCV RNA positive were available for HCV genotyping analysis. Our result demonstrated that there were 4 HCV genotypes and 7 sub-genotypes distribution. The samples were distributed as follows: twenty-five (44.6%) isolated from these patients belonged to HCV-1 [HCV sub-genotype 1a (HCV-1a); 44.0%, 11/25 and HCV-1b; 56.0%, 14/25)], while 21 (37.5%) patients were infected with HCV-3 (HCV-3a; 71.4%, 15/21 and HCV-3b; 28.6%, 6/21). In addition, 9 (16.1%) patients were infected with HCV-6 (HCV-6f; 55.6%, 5/9 and HCV-6n; 44.4%) and 1 (1.8%) individual was infected with HCV-2b.

**Fig. 1:**
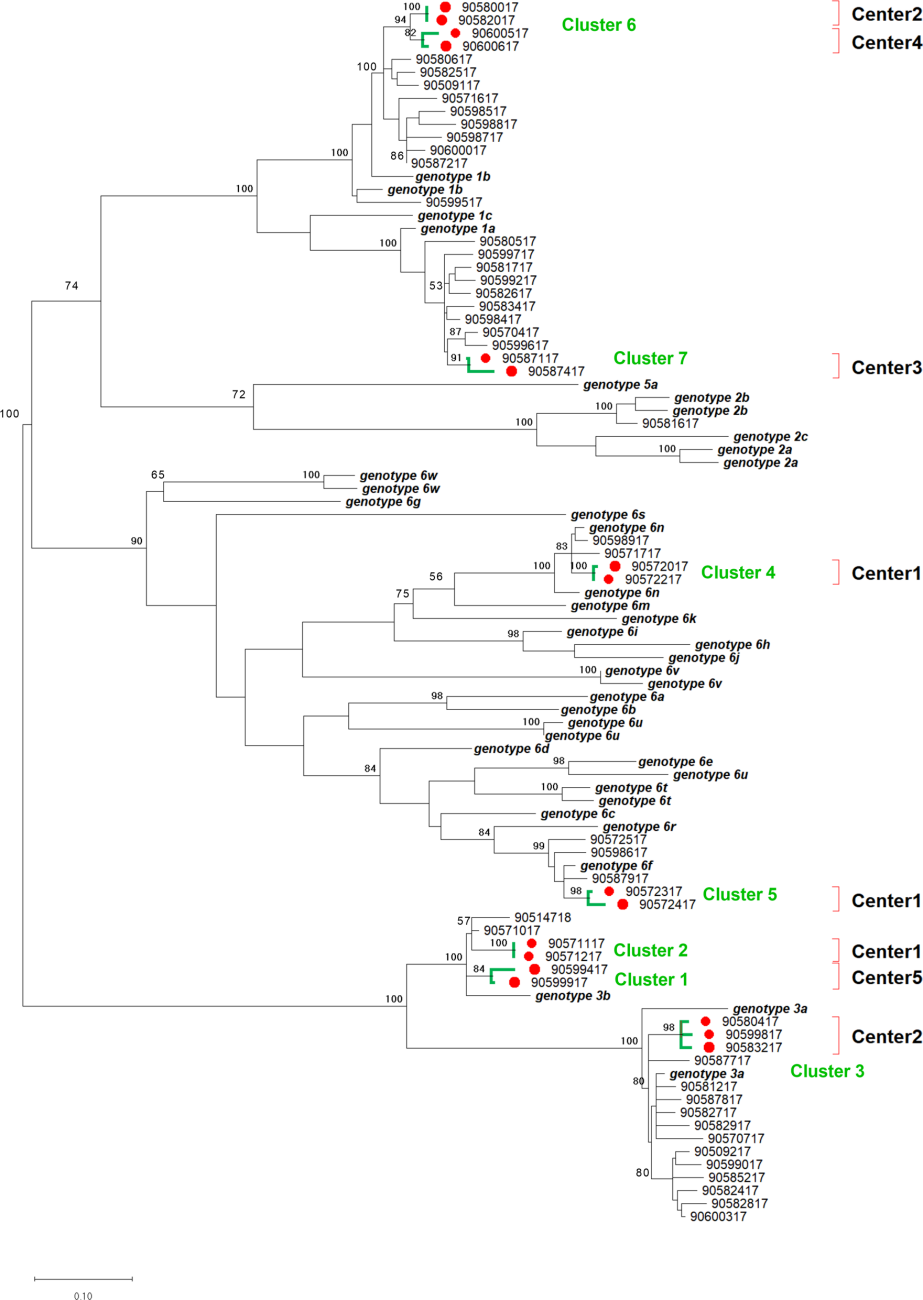
Phylogenetic tree of HCV NS5B sequence based on maximum likelihood using MEGA X version 10.2.1 under maximum-likelihood method with 1,000 replicate bootstrap under the GTR + G + I model. Cluster Picker software identified 7 phylogenic clusters at a branch support threshold of 0.7 and a genetic distance threshold of 0.045 (green branch line and green alphabet), which represented patient-to-patient transmission clusters (17 patients are shown by red circle). Red right square brackets represent each hemodialysis center. Scale bars indicate nucleotide substitutions per site

To address patient-to-patient transmission, nucleotide sequences obtained in this study were aligned with the sequences deposited in GenBank for HCV genotypes. Phylogenetic tree and transmission clusters were also constructed (Fig. 1). The Cluster Picker identified 7 potential transmission clusters of patients undergoing maintenance HD in separate dialysis centers, which defined as monophyletic clusters of two or more patients’ sequence with more than 70% bootstrap support and at genetic threshold of 4.5%. In this study, we found 30.4% of patients (17/56) were suspected to be infected with HCV by using the same HD machines from 5 different HD centers. In center 1, two patients were infected with HCV-6n and clustered to each other (cluster 4) with a bootstrap value of 100**.** Two patients were infected with HCV-6f and clustered with bootstrap value of 98 (cluster 5) and two patients were infected with HCV-3b and clustered with bootstrap value of 100 (cluster 2). In center 2, Two patients were infected with HCV-1b and clustered with bootstrap value of 100 (cluster 6) and three patients were infected with HCV-3a and clustered with bootstrap value of 98 (cluster 3). In center 3, two patients were infected with HCV-1a and clustered with bootstrap value of 91 (cluster 7). In center 4, two patients were infected with HCV-1b and clustered with bootstrap value of 82 (cluster 6). In center 5, two patients were infected with HCV-3b and clustered with bootstrap value of 84 (cluster 1). The phylogenetic tree indicated high homology and close clustering of HCV quasispecies among these cases, patient-to-patient transmission of HCV was highly suggestive in the respective facilities.

## Discussion

Chronic HCV infection constitutes a health issue worldwide due to its adverse impact on quality of life and survival of infected individuals in terms of progressive liver disease and extrahepatic manifestations including CKD [1]. The burden of HCV infection among patients with ESRD undergoing dialysis varies considerably among dialysis units worldwide and its prevalence remains higher than in the general population [5]. Currently, there are limited data regarding the molecular epidemiology of HCV among patients undergoing long-term dialysis in Thailand. In this multicenter study, we showed that the prevalence of anti-HCV antibodies among patients with ESRD was approximately 4.2%, which appeared to be similar to the report of the Renal Registry of Thailand (4%)[19] but was approximately 3 times higher than in the Thai general population with the same age group (1.5%) [3]. Of note, our seroprevalence rate was comparable to those reports in the developed world (1.5%‐30%), but lower than those found in other developing countries with prevalence rates of 5–40% [9]. These data might indicate the different epidemiological characteristics of the respective general populations, as well as various factors that might be associated with inadequate control processes against the infection within dialysis facilities.

Identifying HCV genotype has clinical significance in relation to disease manifestations and treatment outcome. In this study, several HCV genotypes including HCV-1a, -1b, -2b, -3a, -3b, and -6 variants were identified. Of note, the observed predominance of HCV-1 (HCV-1a/1b) differed, to some extent, from the Thai general population that the most common genotype is HCV-3, followed by HCV-1 and HCV-6. In fact, our molecular epidemiological studies showed that HCV-3a continued to be the most common genotype in Thailand ranging from 43.5 to 51.5% over the past decade [3, 20]. Interestingly, high prevalence of HCV-1 in our study was in line with previous reports from several dialysis centers in Asian, European and South American countries [21]. The mechanism by which HCV-1 typically prevails over other HCV genotypes among patients with ESRD is not clear and needs further investigation. Also, it should be mentioned that a high proportion of HCV-6 variants (approximately 20%) in this report was similar to the observation among the Thai general population. Indeed, HCV-6 is distributed predominantly in south China and Southeast Asia and displays marked genetic diversity [2]. For instance, HCV-6 variants were reported in an outbreak from a low-resourced dialysis unit and were also common among patients undergoing dialysis in an epidemiological study in Vietnam [22, 23].

Giving high rates of transmission through parenteral routes, HCV infection in the community is mainly caused by high-risk behaviors, such as injecting drug users (IDU) and sexual practices [24]. In this respect, our previous report demonstrated that a high proportion of HCV mono-infected and HIV/HCV co-infected Thai patients had a history of IDU and being men who have sex with men (MSM)[25]. Previous Asia–Pacific registry data demonstrated that dialysis modality was an independent factor associated with HCV infection as higher rates of anti-HCV positivity were detected in patients undergoing HD compared with PD [26]. These results might reflect the cumulative risk of nosocomial infection over time, particularly in patients undergoing long-term dialysis vintage. Indeed, a recent meta-analysis in Asia populations also demonstrated a positive correlation between duration of HD and the risk of HCV infection [27]. For instance, patients with prolonged HD over 5 years were at increased risk of developing HCV infection six times higher than those with shorter HD duration, which was in line with this report.

The evidence of person-to-person transmission of HCV among patients in our cohort was further supported by phylogenetic analysis of the NS5B region. In this context, several studies showed that phylogenetic analysis of individual HCV RNA isolates could provide valuable information of nosocomial transmission of HCV occurring within dialysis units that might not be assessed through traditional epidemiological approaches [28–30]. Our results identified 7 separate clusters of various HCV isolates involving 17 patients with high sequence homology to each other in distinct dialysis centers. These results emphasized the unrecognized HCV transmission within dialysis units might occur more frequently than anticipated due to asymptomatic or mild hepatitis among most infected individuals.

The KDIGO guidelines strongly recommend strict infection-control procedures in HD facilities, however, nosocomial transmission of HCV has been repeatedly observed, especially in low-, and middle-income countries [31]. Several potential risk factors and practices associated with patient-to-patient transmission have been proposed to explain such an increased risk of HCV infection, including dialyzer reuse, internal contamination of HD machines and contamination by staff members [9]. In this study, it was possible that HCV acquisition through dialysis machinery or by dialyzer reuse might be responsible for the small-scale outbreaks in infected individuals who shared the machine. Unfortunately, the exact mechanism of HCV transmission in each facility remains to be discovered and is currently under investigation. Similar to our report, a recent study from Vietnam demonstrated that sharing dialysis machines and environmental contamination might be contributable to an outbreak of HCV in a resource-limited HD unit [22]. Thus, strict adherence to standard infection control procedures regarding dialyzer reuse, disinfection of machines and hygienic precautions are highly needed [28]. In addition, regular screening for HCV infection is recommended for preventing HCV transmission within HD facilities [32].

The standard assessment for active HCV infection requires screening test with anti-HCV antibodies followed by the confirmation of NAT. However, HCV RNA testing is limited in developing countries due to high costs and the necessity of equipped laboratories. Serum HCVcAg has been shown to be an alternative assay to NAT due to its more practicability and less expensive. In the setting of low- and middle-income countries, World Health Organization (WHO) guidance recommends the utility of HCVcAg as an alternative test to diagnose HCV viremia when NAT is not accessible [33]. In this study, we directly compared the use of HCVcAg and HCV RNA measurement in patients with anti-HCV positivity. Our results showed that HCVcAg assay exhibited good performance and highly correlated with HCV RNA concentrations (r = 0.955, *P* < 0.001). Indeed, the correlation coefficients between the two markers in patients with ESRD were exceeding 0.90 in most previous reports [34, 35]. It should be mentioned that the correlation between both assays appeared to be high among patients with impaired immune response, including liver and kidney transplant recipients and patients with HIV co-infection [36, 37]. Of note, of the three samples in this report that tested negative for HCVcAg, but were HCV RNA positive, all had low HCV RNA levels. This was in line with previous data indicating good correlation between both markers if viral load is greater than 3000 IU/mL, the level of which is typically found in over 95% of untreated HCV individuals [33]. Given its high diagnostic accuracy and feasibility, we propose that HCVcAg testing could be used as a reliable biomarker of HCV viremia in guiding clinical decisions among patients with ESRD, particularly in resource-limited settings, where HCV RNA testing is not routinely available and cost-saving strategy is desirable.

A new paradigm of HCV treatment has improved considerably after the development of DAAs that specifically target viral replication in HCV life cycle [7]. Recent evidence has shown that HCV eradication or SVR can now be achieved in over 90% of patients with late-stage CKD treated with 12-week interferon‐free DAA regimens [38, 39]. Growing evidence also supports the use of sofosbuvir-based regimens, which is safe and well tolerated, resulting in SVR rates of 97% in a meta-analysis of HCV-infected patients with ESRD [40]. Recent data have indicated that successful HCV therapy with DAAs can result in a decrease in liver-related complications and the incidence of HCC, accompanied by an increased in overall survival rates[7]. In this cohort, approximately 10% of patients with HCV infection had significant fibrosis as assessed by APRI score. Without HCV treatment, these patients might eventually develop cirrhosis and liver-related complications. As benefits of HCV eradication with DAAs outweigh potential harm, it is now recommended that all HCV-infected patients with CKD should be evaluated for therapy according to KDIGO 2018 clinical practice guideline [10]. Another advantage of DAA therapy is that the risk of nosocomial transmission of HCV within HD facilities is likely to be reduced following treatment-as-prevention strategy. To this end, micro-elimination of HCV from HD units achieved by the combination of prevention and treatment is now considered as a realistic goal [41].

**Fig. 2:**
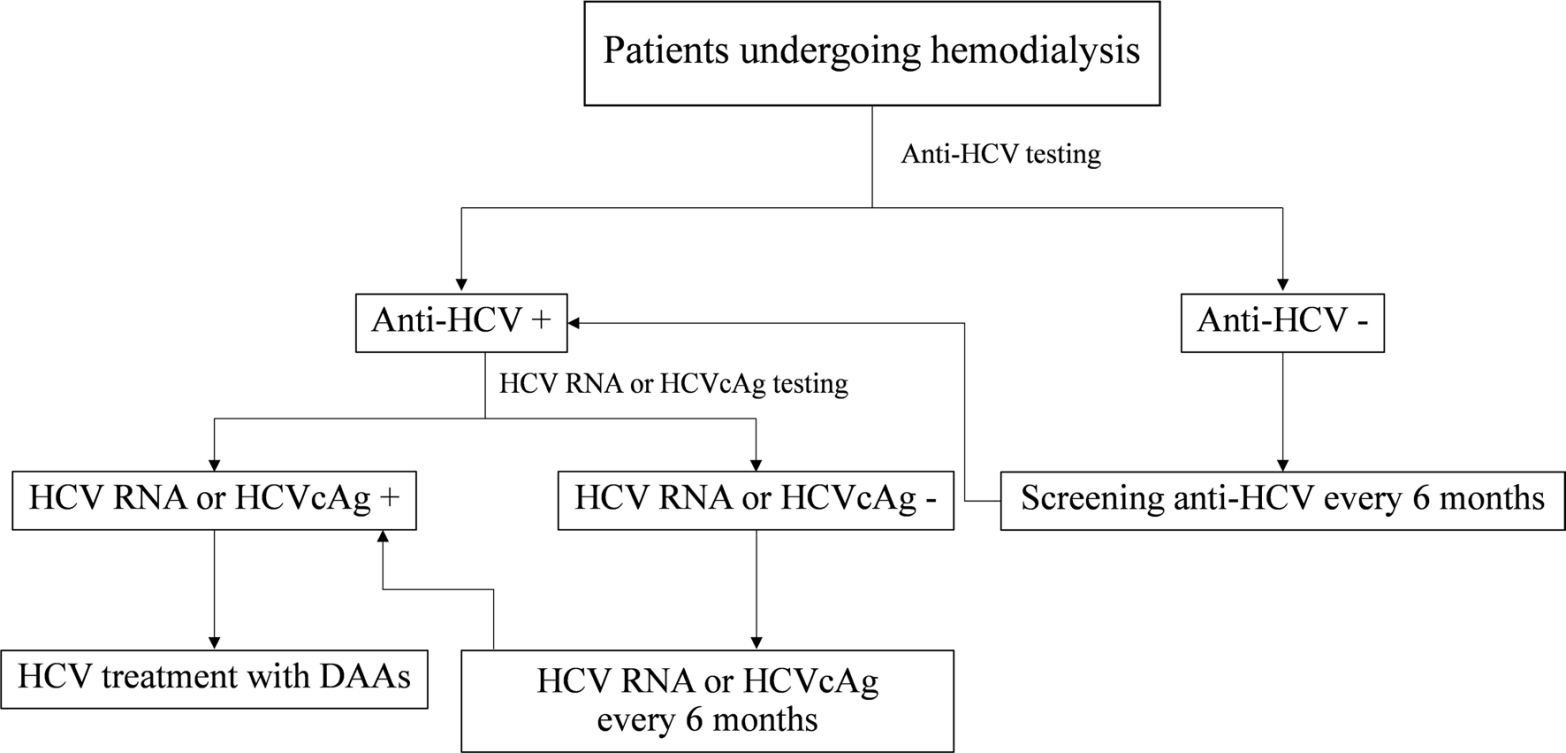
Proposed algorithm for screening HCV infection in patients undergoing hemodialysis

Based on the KDIGO guidelines [31], we proposed a simplified algorithm for HCV screening and management among patients undergoing HD, particularly in resource-limited settings (Fig. 2). Screening with anti-HCV antibodies in patients with HD should be considered upon initiation of HD to document baseline HCV status. Among those with anti-HCV-positive, confirmation of viral replication through HCV RNA testing is required. Alternatively, serum HCVcAg could be used as a substitute test when HCV RNA quantification is not available and/or not affordable [12]. As liver biopsy is associated with an increased risk of procedure-related complications, non-invasive evaluation for staging of liver fibrosis such as serum biomarkers (e.g., APRI score) or liver stiffness measurement is also recommended for HCV-infected individuals [42]. According to the American Association for the Study of Liver Diseases and the Infectious Diseases Society of America (AASLD/IDSA) guidelines, all HCV-infected patients should be considered for DAA treatment except for those with a limited life expectancy (< 12 months) due to liver disease or non-liver-related comorbid conditions [43]. The choice of specific DAA regimens is based on several factors including HCV genotype, prior treatment history, drug-drug interactions and comorbidities. If available, however, it is recommended to select pan-genotypic and ribavirin-free DAA regimens [43]. Patients who are not infected with HCV should be regularly screened for new infection using anti-HCV antibodies every 6 months. Finally, close collaboration between nephrologists and hepatologists is essential for effective diagnosis, prevention and treatment of HCV in patients with ESRD.

Although the practice of dialyzer reuse in developed countries such as in US, European Union countries, and Japan was discontinued, reuse dialyzer in resource-limited countries has been continuing and remains predominant in HD facilities. The need for dialyzer reuse is to balance the financial constrain of national health budget and accessibility of HD for patients with ESRD. The Kidney Disease Outcomes Quality Initiative (KDOQI) recommends that dialysis facilities choosing to reuse dialyzers must follow the Association for the Advancement of Medical Instrumentation (AAMI) recommendations for reprocessing [44]. Although dialyzer reuse has potential for nosocomial transmission of infection in HD facilities, a systematic review of 1176 studies in 956,807 patients demonstrated that there was no significant difference of patient’s mortality between reused and non-reused dialyzer [45]. In Thailand, although there are trends to perform single use dialyzer among HD facilities, reuse of dialyzer is allowed if HIV antibody test is negative. For patients who test anti-HCV positive, the reuse of dialyzer could also be permitted but the reprocessing must be separated in isolated reuse facility and follow the AAMI recommendation. With addition of HCVcAg testing, we suggest that only single use dialyzer should be allowed in patients who test HCVcAg or HCV RNA positive.

There are some limitations in our study. First, we used the NS5B region for assessing HCV genotype distribution. In this context, previous data have indicated that the analysis of only this HCV region provides an accurate characterization of genotypes and sub-genotypes [15, 16]. Second, it could be possible that other risk factors, such as IDU and MSM shared by infected patients, might be responsible for HCV transmission outside the HD centers. Finally, the approach of HCVcAg testing might has a decreased sensitivity in patients with low HCV RNA, which accounted for 5% of individuals in our report. In fact, the probability of HCV contamination is considered to be insignificant in low level viremia. Additionally, HCVcAg quantification must be repeated at least semi-annually in patients with anti-HCV positivity based on our proposed algorithm (Fig. 2). Following this recommendation, the assay will be able to detect active infection if increasing viremia occurs in the natural course of chronic HCV infection.

## Conclusions

This study indicated that HCV prevalence among Thai patients with ESRD remained high and its genotype distribution slightly differed to those of the general population. HCVcAg testing could be an alternative assay to HCV RNA in resource-limited settings. Our data based on phylogenetic analysis provided evidence supporting that patient-to-patient transmission of HCV occurred within HD units and indicate the importance of infection‐control strategies. As patients with ESRD are increased risk of acquiring HCV infection via HD, they should undergo regular screening and monitoring for the infection. In the era of highly effective DAAs, any HCV-infected patients should be considered for viral eradication therapy to reduce the risk of progressive liver disease as well as to prevent further viral transmission.

## Data Availability

The nucleotide sequences of NS5B gene were submitted to the GenBank database under accession numbers MT428260-MT428322.

## Abbreviations

CKD: Chronic kidney disease
ESRD: End-stage renal disease
HCV: Hepatitis C virus
DAAs: Direct-acting antivirals
SVR: Sustained virological response
HD: Hemodialysis
IDU: Injecting drug users
MSM: Men who have sex with men
KDIGO: Kidney Disease: Improving Global Outcome
NAT: Nucleic acid testing
HCVcAg: Hepatitis C virus core antigen
HBV: Hepatitis B virus
HIV: Human immunodeficiency virus
AST: Aspartate aminotransferase
ALT: Alanine aminotransferase
APRI: AST/platelet ratio index
AASLD/IDSA: American Association for the Study of Liver Diseases and the Infectious Diseases Society of America (AASLD/IDSA)
KDOQI: Kidney Disease Outcomes Quality Initiative
AAMI: Advancement of Medical Instrumentation
